# It’s all about relationships: A qualitative study of family physicians’ teaching experiences in rural longitudinal clerkships

**DOI:** 10.1007/s40037-018-0416-y

**Published:** 2018-03-12

**Authors:** Cary Cuncic, Glenn Regehr, Heather Frost, Joanna Bates

**Affiliations:** Centre for Health Education Scholarship, departments of Medicine, Surgery and Family Medicine, University of British Columbia, Vancouver, BC Canada

**Keywords:** Relationship-based teaching, Longitudinal integrated clerkships, Family medicine preceptors

## Abstract

**Introduction:**

The relationship between preceptor and trainee is becoming recognized as a critical component of teaching, in particular in the negotiation of feedback and in the formation of professional identity. This paper elaborates on the nature of the relationships between preceptor and student that evolve in the context of rural longitudinal integrated clerkships (LICs).

**Methods:**

We drew on constructivist grounded theory for the research approach. We interviewed nine LIC family practice preceptors from three sites at one educational institution. We adapted the interview framework based on early findings. We analyzed the data through a constant comparative process. We then drew on concepts of relationship-based learning as sensitizing concepts in a secondary analysis.

**Results:**

We constructed three themes from the data. First, preceptors developed trusting professional and personal relationships with students over time. These relationships expanded to include friendship, advocacy, and ongoing contact beyond the clerkship year. Second, preceptors’ approach to teaching was anchored in the relationship with an understanding of the individual student. Third, preceptors set learning goals collaboratively with their students, based not only on program objectives, but also with the student as a future physician in mind.

**Discussion:**

Our findings suggest that rural family medicine preceptors developed engaged and trusting relationships with their students over time. These relationships imbued all activities of teaching and learning with an individual and personal focus. This orientation may be a key factor in supporting the learning outcomes demonstrated for students studying in rural LICs.

## What this paper adds

Given the documented positive outcomes of longitudinal clerkship placements for students, it behoves us to carefully examine the processes of teaching and learning that may contribute to these outcomes. While we are coming to understand students’ experiences, we as yet know little about how their preceptors teach. Our findings suggest that rural family medicine preceptors develop engaged and trusting professional and personal relationships with their students over time. These relationships imbue all activities of teaching and learning with personal focus. This may be a factor in supporting the learning outcomes demonstrated for students studying in rural LICs.

## Introduction

Longitudinal integrated clerkships (LICs) are being implemented as an educational model for clinical training around the world [1]. Multiple models are emerging that variably enable educational integration, continuity, and longitudinality, which are the hallmarks of LICs [2]. In one common model of LICs, students are placed in small rural communities during their principal clinical year, where they engage in an extended learning relationship with a family practice preceptor and participate in the comprehensive care of that preceptor’s patients over time. Through this extended, integrated experience, they meet the majority of the year’s core clinical competencies across multiple disciplines simultaneously [3]. The many benefits of LICs for students include positive learning experiences, development of professional identity, patient-centredness and at least equal academic performance. These features are well described in the literature [4, 5].

We have less information about the experience of preceptors in LICs. Moreover, papers that do offer a glimpse into the longitudinal teaching experience of preceptors are either focused on specialty preceptors in teaching hospitals [6] or describe the benefits experienced by family physicians in teaching [7, 8]. Thus, we know less about the teaching methods of family medicine preceptors who are the primary (and sometimes only) teachers of the student in rural LIC clerkships. While there is good evidence of equivalent academic outcomes for these students [5, 9, 10], we do not know as much about how rural family medicine preceptors interact with students in longitudinal models of clerkship.

We aimed to add to this limited body of research by exploring family medicine preceptor experiences of student clerkship supervision in LIC rural sites. Our goal was to better understand the mechanisms underlying the enhanced student experiences described in the literature.

## Methods

### Setting

This study took place at the University of British Columbia (UBC). The UBC undergraduate medical program consists of four years of training with the principal clinical clerkship year occurring in year three. Third year LICs were implemented in 2004 [11] based on the Australian model [12] and, at the time of the study, the university had three LIC sites. Preceptors in our study had trained several LIC students, as well as rotational students. All sites were small communities, distant from the main academic institution (Tab. 1), and required the student to relocate to the LIC site. In all sites, a given primary preceptor (a family practice physician) supervised the same single medical student in his or her own clinical practice for at least one day each week over the clerkship year. Students were immersed in the clinical work of the supervising preceptor, including caring for the preceptor’s in-hospital, emergency and obstetric patients as well as patients in the family practice clinic. The students spent the remainder of the week in other clinical activities in the same community to meet the year’s learning objectives. The LIC site directors were responsible for the students’ final summative assessments. Students self-selected the LIC option and spent almost all of the clinical year (42 weeks) with their longitudinal preceptors, integrated into the patient care team of the practice, working with family doctors and (where available) specialists across the healthcare system in their respective communities.

**Table 1:** Description of longitudinal integrated clerkship sites

Site	Distance (km) from closest urban centre	Distance (km) from university	Population	Number of years with LIC students	Number of LIC family practice preceptors	Number of students at site per year
A	100	100	80,000	6	7	6
B	600	1350	11,000	3	3	3
C	450	1200	22,000	2	4	4

### Study population

The study population consisted of the 14 current LIC family physician primary preceptors from all three LIC sites.

### Study design

We drew on constructivist grounded theory (CGT) [13] for our methodological approach. CGT recognizes that the analytic process is shaped by the researcher’s interaction with the study participants, and that meaning is co-constructed by members of the research team. Our study maintained the rigour of CGT in its iterative cycles of data collection and analysis, constant comparison analysis, and the use of memo writing in order to produce an audit trail from data to interpretation.

This study was approved by the research ethics board of UBC.

### Data collection

We invited all 14 family physician primary preceptors from the three sites to participate, and interviewed all consenting preceptors. Due to the small study population, our sample was a convenience sample. Interviews were conducted face-to-face at the participant’s clinical office by a single member of the research team (CC). Interviews were audio recorded and transcribed verbatim. We started with very open ended questions, asking preceptors to describe their experiences with teaching and assessing their students. Consistent with the tenets of Charmaz’s constructivist grounded theory [13], the research team discussed the early interviews and adjusted the interview framework after every three interviews. The interviewer (CC) wrote notes after each interview to capture its tone and the insights that emerged at the time of interview.

### Data analysis

Analysis was both inductive (in that themes were generated from the data) and deductive (in that identified themes were tested through analysis of subsequent data). Two investigators (CC and HF) read three transcripts and independently identified emergent themes. These themes were discussed by the research team (CC, HF, JB) until agreement was reached on an initial set of themes. Two investigators (CC and HF) then coded the remaining transcripts, discussing any discrepant data and reaching agreement on a final coding structure. Memos were used to create an audit trail of theme development. In the second stage of the analysis, the research team (CC, HF, JB, and GR) discussed the meaning of the findings. Each member of the research team contributed to the further analysis, suggested insights based on their own conceptual lens, and identified pertinent literature and theoretical frameworks for consideration. As the analysis progressed, we drew on the concepts of relationship-centred teaching [14] to further interpret our findings. A careful audit trail of progressive insights and the connection of those insights to the coded data and theoretical constructs was maintained throughout the analysis.

### Research team

At the time of the study, the principal investigator (CC) was a general internist at an urban medical school, supervising students and residents during their inpatient and outpatient rotations. She was completing a Masters in Medical Education. The senior investigator (JB) was, at the time of the study, a former preceptor in a community-based family practice setting, a medical education researcher using qualitative methods, and had been involved with the implementation of the LICs. The third investigator (HF) was a socio-cultural geographer, qualitative researcher, and at the time of the study was engaging in a post-doctoral fellowship in Health Professions Education. The final member of the research team (GR) was a cognitive psychologist and medical education researcher with long-standing experience with qualitative research.

## Results

### Participants

Nine of the 14 preceptors agreed to be interviewed for the study. Eight of the recruited preceptors were male and one was female (non-participants included three male and two female preceptors). The number of years in clinical practice ranged from 3 to 35 years. Each preceptor had supervised 1–5 LIC students. Four preceptors had previously supervised medical students in block rotations of family medicine electives and five had only supervised LIC students. Four had experience teaching family practice residents. Seven preceptors were trained in Canada and two were trained overseas. Preceptors spoke freely and openly about their experiences with students. Interviews lasted 37–110 min.

We constructed three themes from the data. First, preceptors developed trusting professional and personal relationships with students over time. Second, the preceptors’ overall approaches to teaching appeared to be anchored in the relationship with, and understanding of, the individual student. Third, preceptors set learning goals collaboratively with the student, based not only on program objectives, but also with the student as a future physician in mind.

### The preceptors’ relationships with their students developed over time

#### Preceptors came to care for their students as people

Preceptors described the evolution of a relationship with their LIC student over the year—an evolution that led to a personal as well as a professional relationship. They came to care about their students as people, they became interested in students’ lives outside of medicine and they became insightful about and respectful of students’ individual learning needs. They viewed their students holistically and were concerned not only about their academic progress, but also about their emotional well-being. Preceptors wanted to know how their students were doing in other aspects of the clerkship beyond their own practice context. Illustrative of this deep relationship, preceptors described conversations with students about their struggles and their personal lives, in which students might ask for advice. As one preceptor elaborated,

*I know when he sees his girlfriend … we talk a lot about personal stuff that you don’t tell anyone else*. (Participant 8)

Relationships developed with elements of friendship, such as actively socializing together through dinners and outdoor activities:

*We [preceptor and student] had a lot in common; her husband was into hunting and fishing, so was she*. (Participant 4)

Moreover, the relationship-building was embedded in the small community where everyone knew each other, so these relationships could be enmeshed in complicated ways. For example, one preceptor’s spouse made plans to go jogging with an LIC student based in the community, who was also a classmate of the preceptor’s student. Because of these elaborated relationships, preceptors commented that it was difficult (‘wrenching’—Participant 5) to say goodbye at the end of the academic year. Some of these friendships persisted beyond the clinical year with the preceptors staying in touch with their students as friends. Even the one preceptor who explicitly disagreed with this notion of developing a personal relationship with the student (‘*I’m not the social chitty-chatty type and I think they know that … I’m not their confidante … I’m their preceptor*’) nonetheless stated later in the interview that his students have

*multiple interactions, with your kids and your family because they—you’re obviously socializing with them in various things*. (Participant 6)

#### The preceptor–student relationship enabled trust to develop

Preceptors explained that they worked closely with students, sharing emotionally draining experiences such as births, deaths and breaking bad news, and debriefing together after these experiences. As one preceptor articulated:

… *there’s a huge trust component that goes on between the two of you … you share a lot of life experiences that can be pretty earth-shattering.* (Participant 5)

Preceptors elaborated that trust was built gradually and reciprocally between themselves and the student. They described ‘deep’ relationships compared with their experience of relationships with students on 4‑week family medicine rotations.

#### Caveats to the longitudinal relationship

Some preceptors spoke of the need to maintain professional boundaries while enabling this closeness to develop. They explained that in smaller communities, family physicians cannot help but see patients socially: through these experiences, the physicians became aware of juggling their different roles. With the student, the preceptors also moved back and forth between listening to personal quandaries, giving corrective feedback, providing formal direction, and so on:

*I struggle with that boundary, where we should be at, but I try to—but I think they’re okay with that, too, because if they blow a case or don’t do something right, I still can give them constructive criticism on what they should be doing. And they—when you’re in the office setting, the hospital setting, they—you put on your different hat, basically, and the students put on their different hat, too. So I think they’re okay with that. The feedback I get from the other preceptors, especially the residents, they’re all kind of social with their preceptors anyways. *(Participant 3)

While preceptors described mostly positive experiences, they identified the ‘struggling student’ as something that required a lot of effort. One preceptor elaborated on the effects of having an academically struggling student for an entire year:

*He couldn’t narrow his net; he’s cast his net way too wide for problems. It was painful, patients came out saying, ‘I’m done’ they don’t want to see him again because it took so long. It was horrible for the practice. *(Participant 3)

The effects of a challenging student were felt by more than just the primary preceptor.

*That made it hard on everybody, and it’s amazing how one person can make it difficult for everybody. For all the preceptors.* (Participant 5)

Preceptors spoke of these struggling students as a challenge for the entire teaching community, in part because they accessed peer and site director support for themselves as well as their student. In spite of the difficult teaching situation, they did not seem to lose their empathy for the student. They continued to speak of the student with compassion, although no preceptor spoke of a close social relationship with a student who was struggling academically. However, even preceptors who had not directly experienced a challenging student spoke of the fear that a ‘bad student year’ would result in their becoming ‘burned out’ (Participant 9).

#### Students viewed as junior colleagues

Preceptors considered their students not only as students who would move on to the next level of training, but also as future colleagues. Some students stood out as clinically exceptional, and preceptors spoke of these exceptional students as ‘a colleague without the experience’ (Participant 1), and gave examples of learning from these students. As one preceptor stated:

*My last year’s student was absolutely phenomenal, so I can’t really compare anyone to her. It was—she was stellar in everything … I couldn’t teach her anything. She taught me probably.* (Participant 3)

### Preceptor approaches to teaching were anchored in their relationships

The preceptors’ approaches to teaching were influenced by and anchored in their longitudinal supportive relationships with their students.

#### Preceptors created a positive learning environment

Preceptors remembered their own experiences as students navigating the clinical year, and empathized with their students:

*I think it’s hard once you get in to recognize there’s a big transition from starting medical school thinking you’re going to be this person like on TV and save these lives and getting there and realizing that’s really not necessarily the reality.* (Participant 2)

Thus, preceptors described making deliberate efforts to create a positive learning environment by being friendly, welcoming, slowing down their clinic for students so as not to overwhelm them, and taking interest in the student as a person. Preceptors were especially careful to be supportive when providing feedback, although they felt comfortable as the relationship developed to give clear and corrective feedback. The preceptors described such feedback as a central teaching strategy that promoted student learning. And thus approached giving feedback as working to improve the students’ abilities rather than judging them. They felt that the trusting relationship with the student could weather even challenging feedback and difficult circumstances and that the close relationship with the students in fact facilitated constructive feedback because it came from a place of caring about the student:

*… maybe like almost a marital relationship, you know? Where you’re more likely to say to your partner, ‘I love you to pieces, and, but this is an aspect of you that you know can sometimes rub other people the wrong way.’ *(Participant 1)

While preceptors spoke comfortably about giving their students feedback, they did not see assessment as their primary role. The site directors were responsible for the summative assessments of the students.

#### Preceptors tailored their teaching to the student

In addition to creating a supportive learning environment, preceptors described other teaching strategies that they used with each individual student. They selected patients for the student carefully in order to maximize their learning without overwhelming them. They negotiated with students to tailor the learning experiences, not only in terms of difficulty but also in terms of content. Preceptors viewed students as unique individuals with their own goals and learning objectives. For example, if students had particular interests, preceptors encouraged them to pursue those interests by providing appropriate patients:

*She’s got an orthopaedics exam coming up at the end of the month so we’re focusing on orthopaedics. She identified a personal need for more paediatrics so we’re looking at the kids as well. So it’s mainly driven by her agenda. But it’s also driven by mine, to some extent. *(Participant 9)

The preceptors’ strategies to enhance their students’ learning went beyond teaching within their own practices. They explained that rural communities are by nature tightly connected communities socially and professionally. The preceptors leveraged their professional networks to enable their students to learn beyond their own practices:

*I have a colleague who only does female health and I’ve asked her to get my student involved*. (Participant 8)

#### Preceptors acted as student advocates

Grounded in the relationship, preceptors took on a role not only as a confidante, but actively taking steps to help students with their struggles that were occurring outside of their own clinic. In this way they acted as student advocates. For example, one student had a minor physical disability which interfered with his performance in the operating room, leading to poor evaluations from the surgical preceptor. The family practice preceptor discussed the condition with the medical student, and as a result the student started taking medication, with positive results. Another example of advocacy arose when a distressed student shared her personal struggles with her preceptor, who then supported her through negotiation with the site director.

*Finally on the one Friday I said, ‘Are things getting better?’ And she just said, ‘No,’ and she’s crying. I said, ‘Well, look, we’ve got to go and talk to the director.’ So I went down with her … she just really was struggling.* (Participant 2)

At times this support lasted beyond the clerkship year. One preceptor described talking with and supporting a former student who was struggling in their residency program.

### Preceptors’ goals for student learning beyond program objectives

Whether students were exceptional or arrived with noticeable gaps, preceptors set goals for their students that went beyond the program objectives.

#### Preceptors negotiated learning goals with their student

Preceptors viewed the clerkship as a social process, in which the preceptor and the student worked together side by side: learning goals emerged from this collaborative practice. On a daily basis, preceptors judged how well their students performed on clinical tasks. They gave feedback and then set learning goals collaboratively with the student. Preceptors increased student responsibility as performance developed, leading to the setting of new learning goals. Preceptors felt pride in their students’ progress in performing clinical tasks. Learning goals also included the development of good relationships with patients.

*If you’ve been there long enough you certainly do get to know your patients. And I think the students get to feel that.* (Participant 4)

Preceptors’ learning goals for students extended beyond their own clinical practices. They described how students confided their academic struggles, weaknesses and insecurities that they were experiencing beyond their preceptors’ clinics.

*He [student] struggled not just with general practice, with surgery, obstetrics, all—anaesthesia, elective, all that, it was the same … because I knew he was struggling, I went back to basics. *(Participant 3)

As the preceptors came to know their students better, they negotiated learning goals with the student to encourage unique interests. Students who were interested, for example, in obstetrics as a career were encouraged and enabled to set and meet learning goals that were beyond the usual level of clerkship.

*She was also interested in doing [specialty] so I phoned up our [specialist] and said, ‘Look, would you take her and have her spend some time with you.’ *(Participant 2)

#### Preceptors wanted students to have a realistic view of the profession

Preceptors wanted students to not only improve their clinical performance but also to understand the rewards and realities of practising medicine. As one preceptor elaborated:

*I think probably one of my proudest times is I had one [student] that—said, ‘I’ve worked with other doctors before but I really can see that you love what you do and you love your patients and they love you.’* (Participant 2)

Several preceptors described sharing the challenge of being wrong or of not knowing everything with students. Preceptors indicated comfort with, and a desire to, reveal their own limitations to students, explaining that this was an important learning experience for students. Participant 4 explained,

*that’s an important message: you cannot know everything … you can’t always make a diagnosis.*

#### Learning goals included the student’s personal growth

Furthermore, preceptors expected their students to grow not only in clinical expertise but also in personal maturity. They wanted students to develop their own voice, become more confident as people, overcome personal struggles, and find career paths that would make them happy:

*I’ve really come to realize that I really want to see them sort of sense what they would like to do.* (Participant 1)

#### Preceptors developed teaching philosophies

In these ways, preceptors developed their own philosophies of teaching over time. None of the preceptors claimed that their self-identity had shifted since becoming an LIC preceptor; however, they viewed it as another facet to their profession. They were able to clearly articulate the beliefs they developed about their teaching styles:

*I’m a big believer in that they should be doing it, rather than just observing, they should be taught to do it and coached through it. There’s nothing like doing it yourself. *(Participant 3)

This teaching approach linked to their view of student assessment. They described student assessment in terms of the students being given progressively more challenging tasks. As preceptors concluded that their students mastered one task, they then allowed them to perform that task independently but moved them to start to learn a more difficult task. Preceptors described their assessment processes as intrinsic to the ongoing supervision and progress of their students.

## Discussion

Our study focused on rural family medicine preceptors’ experiences with students attached to them for a year-long clerkship. Some of our findings are aligned with other reports in the literature: our preceptors felt rewarded by their students’ progress [6, 7], strengthened by the learning they themselves accomplished [6], and supported by student companionship [15]. The participants in our study were confident delivering clear corrective feedback and setting learning goals to increase their students’ participation in patient care. Both are hallmarks of effective clerkship supervision [16, 17].

In addition, in this study we were struck by the constant focus of the preceptor on the developing relationship with the student, and the affordances of that relationship. Because of the trust that developed over time, preceptors described opportunities for advocacy, for understanding and addressing student difficulties, of supporting progression of independence, and of collaboration in negotiating learning goals. Furthermore, several of our study participants had also precepted students for shorter rural family medicine blocks, and were able to reflect on the different experiences with students between the two, expanding our understanding. We found that preceptors in a year-long LIC established close relationships with students, saw the student as existing beyond the boundaries of the academic preceptor-student interaction, and supported and enabled growth. This student growth developed along personal as well as professional trajectories. These teaching and learning trajectories enabled by family physician preceptors and created through relationships have been described elsewhere [18]. Those participants who had precepted students for shorter blocks reflected that this expanded and deeper relationship was enabled by the length of the clerkship.

We do not assume that this relationship development is necessarily a positive force in all student experiences. Indeed, readers may respond to some of the descriptions of ‘friendship’ and ‘social activities’ with concern and anxiety about professional boundaries. However, rural physicians are adept at negotiating complex boundaries that shift between friendship and physician-patient relationships [19]. The interactions between student and preceptor in small rural communities are necessarily multifaceted, especially when the engagement is longitudinal and immersive. Unlike others’ experience [20, 21], preceptors in our study were comfortable shifting between personal and professional relationships with students, and between evaluating and coaching them.

Although preceptors were comfortable with shifting boundaries, we do not know how the students themselves responded to the inherent ambiguity in their relationships with their preceptors. Others have suggested that learners can be particularly sensitive to role conflict between assessing and coaching in their preceptors [22]. In our study setting, the preceptors were responsible for formative assessment and feedback encompassed in clinical supervision, but site directors were responsible for final summative assessments. This division of assessment responsibility in our setting may have mitigated such student concerns in our setting, where LIC students have described critical feedback as supportive in the context of a caring preceptor [23].

Beyond assessment and feedback, LIC students have described the collaborative relationship with their preceptors as a supportive and caring partnership based on mutual goals [23–25]. Students felt that the relationship motivated them and enhanced their learning: they appreciated the investment made in them by their preceptors. They said that preceptors’ efforts to know them as individuals including in their personal life augmented the relationship [24]. Trust and respect on the part of the preceptor appeared foundational for students to feel comfortable expressing their uncertainties with clinical medicine [23].

Arguably, having and developing relationships is crucial in the learning process. Relationships have been shown to underpin effective clinical supervision [16], influence the amount of teaching that takes place [26], affect the usefulness of feedback [27–29], contribute to effective working relationships between peers [30] and contribute to professional identity formation [31]. These findings across domains in medical education lend evidence to the importance of affective relationships with students as a key factor to learning in clinical years. As such, we consider that one mechanism for the positive learning outcomes in LICs may rest on the strength of the relationships with students reported by our participants. While preceptors described mostly positive relationships with students, we recognize that some relationships could encompass interpersonal difficulty that could lead to ongoing distress for students in a longitudinal placement. Furthermore, some students posed difficulties for the preceptors, and the burden of a struggling student was exacerbated by the placement being for a year.

Our preceptors described standard activities of precepting such as teaching, assessing and giving feedback. However, they also described acts of mentoring, providing friendship and engaging students in social and professional networks. In urban tertiary care settings, these various roles are distributed amongst a number of preceptors and beyond the program itself; this may lead to no one individual experiencing the student as a complete person. Epstein [32] explains that collaboration, coaching, advocacy, and exploring feelings were all ways in which community-based physician preceptors created a safe learning environment for students.

### Study limitations

We recognize that this was a small study, set in one medical school. However, our participants were varied and were drawn from three different sites. Participation in the study was voluntary, and may have attracted preceptors with positive experiences. In addition, the students were self-selected to this model of clerkship. Further, we caution against transferring our findings to other LIC models and settings: Canadian family practice is focused on longitudinal relationships with patients. This may have influenced both our preceptors, as well as our own perceptions of preceptors’ experience. Across Canada [33], the USA [34] Europe [35], and beyond, the discipline of family medicine places the longitudinal relationship with the patient at the centre of the provision of care. The orientation of preceptors we uncovered in our study toward student centredness may arise from the family physicians’ professional discipline. Documents describing family medicine [36] include such statements as: ‘develops a person-centred approach; promotes patient empowerment; and establishes a relationship over time’. Family physicians themselves articulate this longitudinal relationship as a key professional attribute [33]. We hypothesize that this orientation to relationship-based care may naturally engage the family practice preceptor in relationship-based teaching, thus potentially reducing the transferability of our findings to preceptors drawn from other disciplines. Furthermore, preceptors in our study may have been recruited to teach because they are perceived as caring individuals. Family physicians in Canada are trained to be patient-centred [37]. Three key tenets of patient-centredness [37] resonate with the relationships our preceptors had with their students. These are: seeing the patient [student] as a person, sharing power and responsibility, and developing a therapeutic [educational] alliance. Preceptors in our study developed holistic views of their students, negotiated shared goals, and developed trusting relationships. These activities may have drawn upon (Canadian) family physicians’ expertise in delivering patient-centred care to their patients. Finally, we would note that we were unable to separate out the effects of being a family physician preceptor from the effects of being a rural preceptor.

Because our study was based in a rural setting, we are unable to transfer these findings to urban LICs. The extent to which the geographical setting and the rural community experience influenced the development of relationships is unknown.

### Implications for research and practice

We would welcome investigation of preceptor experiences in other settings, including urban LICs and other countries to examine whether our findings are specific to Canada and we call for further studies to disentangle the threads of family physician or rural impact on medical students’ learning, and to differentiate family physicians teaching in longitudinal models from that of other disciplines. Furthermore, this study did not investigate the impact of teaching on the physician’s professional identity. As clinical teaching engages more community-based physicians, this experience will become a question to address.

Although this was an exploratory study, it has immediate implications for faculty development for preceptors teaching in longitudinal models. Most faculty development for community preceptors focuses on basic skills of clinical supervision and delivering feedback. However, our findings suggest that LIC preceptors may need support in sorting out the fluid nature of the relationship, the boundary setting with their students, and ongoing support with challenging students. Medical education programs more broadly might encourage the development of supportive preceptor-student relationships through seeking out caring, empathic physicians as preceptors and articulating the importance of these relationships in clinical supervision.

## Conclusions

Given the positive outcomes of LICs for students, it behoves us to carefully examine the experiences of preceptors and their teaching processes that may contribute to these outcomes. Our findings suggest that rural family medicine preceptors developed engaged and trusting relationships with their students over time. These relationships imbued all activities of teaching and learning with an individual and personal focus. This orientation may be a key factor in supporting the learning outcomes demonstrated for students studying in rural LICs.
